# Magnetic resonance imaging after most common form of concussion

**DOI:** 10.1186/1471-2342-9-11

**Published:** 2009-06-17

**Authors:** Harald Schrader, Dalia Mickevičiene, Rymante Gleizniene, Silvija Jakstiene, Danguole Surkiene, Lars Jacob Stovner, Diana Obelieniene

**Affiliations:** 1 Department of Neurology and Clinical Neurophysiology, Trondheim University Hospital, 7006 Trondheim, Norway; 2 Department of Neurology, Kaunas University of Medicine, Kaunas, Lithuania; 3 Department of Radiology, Kaunas University of Medicine, Kaunas, Lithuania

## Abstract

**Background:**

Until now there is a lack of carefully controlled studies with conventional MR imaging performed exclusively in concussion with short lasting loss of consciousness (LOC).

**Methods:**

A MR investigation was performed within 24 hours and after 3 months in 20 patients who had suffered a concussion with a verified loss of consciousness of maximally 5 minutes. As a control group, 20 age- and gender matched patients with minor orthopaedic injuries had a MR investigation using the same protocol.

**Results:**

In a concussion population with an average LOC duration of 1. 4 minutes no case with unequivocal intracranial traumatic pathology was detected.

**Conclusion:**

An ordinary concussion with short lasting LOC does not or only seldom result in a degree of diffuse axonal injury (DAI) that is visualized by conventional MR with field strength of 1.0 Tesla (T). Analysis of earlier MR studies in concussion using field strength of 1.5 T as well as of studies with diffusion tensor MR imaging (MR DTI) reveal methodological shortcomings, in particular use of inadequate control groups. There is, therefore, a need for carefully controlled studies using MR of higher field strength and/or studies with MR DTI exclusively in common concussion with LOC of maximally 5 minutes.

## Background

After concussion (or mild traumatic brain injury (MTBI)), a significant proportion of subjects report persisting symptoms that include headache, cognitive dysfunction, dizziness, fatigue, and irritability. This cluster of rather non-specific symptoms has been termed as the postconcussion syndrome (PCS), a condition that has been debated since the end of the 19^th ^century and still remains controversial. Several authors suggest that the symptoms of postconcussion syndrome are due to structural abnormalities and/or organic cerebral dysfunction [1,2]. Others have assumed that the PCS is psychogenic in origin or have proposed a biopsychosocial model to explain its development into chronicity in some individuals [3,4].

For the concept of PCS being caused by an organic injury it seems necessary to assume that the concussion produces long-lasting morphological and/or functional lesions. This is, however, still equivocal, at least when using neuroimaging procedures such as cerebral computer tomography (CT) or magnetic resonance (MR) imaging. Studies using CT show lesions only in a small minority of cases, whereas conventional MR studies show pathology in the acute state in 10 to 57% of cases [5-9]. All these studies included patients that had loss of consciousness (LOC) of up to 15–20 minutes. However, in the most common form of concussion, LOC lasts only from a few seconds to few minutes [10]. Therefore, we wanted with a rigidly controlled design to study whether there may be cases with visually detectable and unequivocal traumatic lesions in conventional MR even if one restricts the investigation to only include cases of concussion with LOC of less than 5 minutes.

## Methods

### Study group

Since traumatic white matter lesions may be confused with foci of non-specific high signal intensity that is associated with normal aging [11,12], the study group was restricted to individuals aged 18 to 40 years. Twenty individuals with concussion and short-lasting LOC who attended the emergency unit at the Kaunas Medical University Hospital during the duty of the participating investigators were consecutively identified if eligible according to strict inclusion and exclusion criteria. By thorough examination of witnesses it was in all cases assured that the LOC did not exceed 5 minutes. Additionally, inquiry was made about presence and duration of retrograde amnesia, anterograde amnesia and confusion. No patient was included in whom a reliable witness report could not be obtained.

The patients had no other injuries than the concussion, except for small skin lesions, bruises and other insignificant injuries. Exclusion criteria were diabetes, hypertension, affective disorders, prior history of alcohol or drug abuse, prior history of psychiatric or neurological disorder, prior history of epilepsy or seizure associated with the concussion, earlier concussion and concussion due to assault. There were also no focal neurological signs and normal neurological status at admission except for amnesia and slight and transitory confusion.

### Control group

As controls, individually sex- and age (plus/minus 3 years of age) matched subjects aged 18 to 40 years with the same exclusion criteria as the study group were taken from the records of the traumatology department by identifying patients with minor to moderate non-head injuries who had attended this department in a time period of maximally 2 weeks after the concussion date of the traumatized matching patient. If there were several potential controls within this period, the patient was taken who concerning the time of the attendance to the clinic was nearest that of the head-traumatized individual.

### MR imaging

After inclusion, the patients with concussion and non-head traumatized controls underwent an MR investigation within 24 hours after the trauma and after 3 months using the same protocol each time. MR imaging was performed on a Gyroscan T10-NT 1.0 T (Philips Medical System). A scout sequence was used to align the subsequent scans. The matrix in all sequences was 205 × 256. Axial imaging plane and 5 mm slice thickness was used for all patients in all sequences. Three pulse sequences were included: T1W/SE (TR – 586 ms, TE – 14 ms; NSA 2); T2W/FLAIR (TR – 5000 ms, TE – 80 ms, TI – 1900 ms, NSA 1); and to detect hemosiderin deposits T2W/FFE (TR – 713 ms, TE – 21 ms; flip angle 15.0, NSA 2). The MR covered the whole brain. The scan time was 15 minutes and the slice number 22.

Final evaluation of MR was made by two neuroradiologists blinded for the diagnostic status of the individual.

The study was in compliance with the ethical principles for medical research involving human subjects according to the Helsinki Declaration and was approved by the Ethical committee of the Medical Faculty of the University of Kaunas. All patients gave written informed consent to participate.

## Results

### Study group and control group

Of the twenty patients in the study group 10 were females (mean age 22.8 years; range 18 to 32 years) and 10 males (mean age 26.5 years; range 18 to 37 years) (see table 1). The concussion was in 12 cases due to a car accident, in 6 cases due to fall, in one case due to collision with a moving machine and in one case due to sporting activity. Duration of LOC was on average 1.4 minutes (range: 0.5 – 5 minutes). Mean duration of retrograde amnesia was 1.4 minutes (range: 0 – 10 minutes), of anterograde amnesia 4.3 minutes (range: 0 – 20 minutes) and of confusion 7.3 minutes (range: 0 – 30 minutes).

**Table 1:** Clinical characteristics and cerebral MR after concussion

Gender	Age	Mechanism	Unconsciousness (min)	Retrograde amnesia (min)	Confusion (min)	Anterograde amnesia (min)	Cerebral MR within 24 hours after trauma	Cerebral MR 3 months after trauma
F	18	MVA	2	5	15	0	NP	NP
F	18	Fall	1	0	0	0	NP	NP
F	19	MVA	0,5	0	10	5	NP	NP
F	19	MVA	2	0	0	0	NP	NP
F	20	Sporting	1	0	0	0	NP	-
F	22	MVA	0,5	3	0	1,5	NP	NP
F	25	Fall	0,5	0	5	0	Subependymal periventricular heterotopia	Unchanged
F	27	MVA	1	0	0	3	NP	NP
F	28	MVA	2	0	30	20	NP	-
F	32	Fall	0,5	0	10	5	NP	NP
M	18	MVA	1	5	0	15	NP	NP
M	18	MVA	1	0	0	0	NP	NP
M	21	MVA	1	0	15	5	NP	NP.
M	21	Fall	3	0	0	0	Nonspecific subcortical focus in left frontal region	Unchanged
M	26	Moving machine	1	0	10	5	NP	NP
M	29	Fall	1	0	0	0	Few nonspecific subcortical hyperintensive T2W/FLAIR foci in frontal regions	Unchanged
M	31	Fall	1	0	5	2	Ependymal cyst in left lateral ventricle	Unchanged
M	32	MVA	5	0	10	10	Multiple nonspecific subcortical hyperintensive T2W/FLAIR foci in both fronto-parietal regions	Unchanged
M	32	MVA	3	5	5	0	NP	NP
M	37	MVA	0,5	10	30	15	NP	NP

MVA: motor vehicle accident NP: no pathology

Of the controls, the 10 females had a mean age of 22.9 years (range 18 to 32 years) and the 10 males a mean age of 26.0 years (range 18 to 39 years) (see table 2). The non-head injuries were in 11 cases contusions in the extremities or of the back, in 3 cases distorsions in the ankle or knee, in 3 cases skin lesions and moderate wounds in the remainder (see table 2).

**Table 2:** Cerebral MR after minor non-head injuries

Gender	Age	Trauma	Cerebral MR shortly after trauma	Cerebral MR 3 months after trauma
F	18	Elbow contusion	NP	NP
F	19	Hand contusion	NP	NP
F	19	Hand contusion	Pineal cyst	Unchanged
F	19	Hand contusion	NP	NP
F	20	Knee contusion	NP	NP
F	23	Ankle distorsion	NP	NP
F	25	Leg wound	NP	NP
F	26	Scratch knee cap	NP	NP
F	28	Knee contusion	NP	NP
F	32	Finger cut	NP	NP
M	18	Abdominal wound	NP	NP
M	19	Leg contusion	NP	NP
M	20	Ankle contusion	NP	NP
M	20	Back contusion	NP	NP
M	26	Finger cut	NP	NP
M	27	Leg wound	Nonspecific T2W/FLAIR hyperintensive focus in left peritrigonal white matter	Unchanged
M	30	Ankle distorsion	NP	NP
M	30	Hand contusion	NP	NP
M	31	Ankle distorsion	NP	NP
M	39	Knee contusion	NP	NP

NP: no pathology

### MR imaging

There was complete inter-rater agreement both for the study group and for the control group, possibly due to the low incidence of pathological findings. MR shortly after the concussion showed no intracranial pathology in 15 patients and was still without pathology after 3 months (see table 1). In particular, there were no visible signs of diffuse axonal injury or micro-hemorrhages. In two of these patients a second MR investigation after 3 months could not be performed due to technical problems. One patient had multiple unspecific high signal intensity foci in both fronto-parietal regions. Another patient had a few hyperintensive foci in both frontal regions, and a third patient a hyperintensive focus in the left frontal region. All three patients were male. The hyperintensive foci were unchanged both concerning number and size in the MR after 3 months. An incidental finding was an ependymal cyst in left lateral ventricle in one patient and a subependymal periventricular heterotopia in another patient.

MR in the control group shortly after the trauma and 3 months later showed no pathology in 18 patients (see table 2). One male control had a nonspecific hyperintensive focus in the left peritrigonal region and another control a pineal cyst.

The prevalence of cases with hyperintensive focus/foci of the control group (n = 1) was not significantly different from that in the study group (n = 3) (p = 0.60; two sided Fisher's exact test)

## Discussion

In the present study in which the most common type of concussion was investigated by MR imaging and inclusion of age-and gender matched non-head injured controls, no case was detected with unequivocal intracranial traumatic pathology. There were three male subjects in which high signal intensity foci were found in the white matter. The changes had the same appearance 3 months post injury and were, therefore, likely to be pre-existing findings of non-traumatic origin. In the control group only one individual had a non-traumatic, pre-existing hyperintensive focus. Possibly due to the low power of the study, the difference in prevalence of pre-existing hyperintensive foci was insignificant. Nevertheless, this result may indicate that individuals prone to head injury differ from healthy individuals and individuals prone to orthopaedic injuries in respect to pre-traumatic MR findings.

The study had the limitation that it was performed with 1.0 T MR and 5 mm slices and that traumatic lesions such as small sub-cortical hemorrhages shown by high field MR may have escaped detection. On the other hand, diffuse axonal injury can be demonstrated with MR of even lower field strength than in the present study [13].

A general methodological problem for earlier studies on concussion with structural neurodiagnostic techniques performed in the last 25 year is that many of them did not have a control group and in those who had, the control group was mostly inadequate. An adequate control group should be age- and gender-matched individuals with minor non-brain injuries such as in the present study. Presumably, such group would have a socio-economic status as well as a degree of accident-proneness, psychopathology and alcohol consumption that is more similar to individuals with concussion than a control group of healthy individuals. It is known that psychopathology may predispose to injuries [14,15] and there are more pathological MR findings in people with psychopathology than in healthy individuals [16-18]. Due to the lack of an adequate control group many earlier studies did not provide convincing evidence that the lesions shown really were of acute traumatic origin. Traumatic white matter lesions may be confused with the nonspecific white matter high signal intensity foci associated with normal aging, hypertension [11,12] and affective disorders [19-21].

There are few earlier studies (field strengths from 0.064 T up to 1.5 T) that selectively have investigated MR findings in concussion [5-9], and no study could be found in which exclusively the most common form of concussion has been studied, i.e. in those who had head traumas with LOC lasting maximally a few minutes. There were also inconsistent and inconclusive results as well as methodological shortcomings such as lack of or inadequate control group.

Taken the results and the mentioned methodological shortcomings of these earlier studies into account and in view of the results of the present study it seems justified to conclude that until now there is no reliable documentation that an ordinary concussion with short lasting LOC in a significant number of cases causes a degree of diffuse axonal injury that is visualized by conventional MR with a field strength up to 1.5 T.

In recent years, several attempts have been made to detect signs of axonal injury by use of diffusion tensor imaging (DTI) MR, a method that is able to demonstrate white matter microstructure deficits

The findings in MTBI patients of these studies were usually discrete, inconsistent and diverging [22-28]. In one recent DTI tractography study of the corpus callosum in mild TBI 1 to 6 days post injury in 10 adolescents with LOC up to 10 minutes and 10 matched uninjured controls, fractional anisotrophy was increased and diffusivity decreased suggestive of cytotoxic edema, i.e. no sign of axonal injury [27]. None of the studies included sub-group analysis of patients with very short lasting LOC. As the size of the heterogeneous samples of patients with concussion generally was small, it is questionable whether any difference between findings in common concussion and controls would have reached statistical significance if only patients with short lasting LOC had been evaluated. An additional important source of error was that the included control groups usually consisted of non-injured healthy individuals. Since there is a link between alcohol consumption and injuries [29], concussion populations most likely have higher average alcohol consumption than non-injured controls. Alcohol abuse is, on the other hand, associated with white matter microstructure deficits shown by DTI in several regions of the brain, between others in areas prone to axonal injury after brain trauma [30-33].

## Conclusion

Using a rigidly controlled design with non-head injured controls and performing a baseline scan within 24 hours followed up with the same protocol at 3 months, this 1.0 T MR study shows that none of the patients with common concussion developed abnormalities that could be clinically (visually) detected and related to the brain trauma. Hence, no support was found for a potential organic substrate for PCS at this level of detection.

Obviously, the study does not rule out subtle traumatic abnormalities below the present detection threshold. In order to demonstrate such discrete traumatic changes (for example axonal injury) with conventional MR (or MR DTI) one should perform studies in a greater MTBI population than have been done hitherto and investigate only patients with common concussion (i.e., with LOC below 5 minutes), preferably using MR with a higher field strength (e.g., 3 T or more). Such studies should include a carefully matched control group of a sufficient size consisting of persons with a similar risk profile for MR changes, e.g. non-head injured individuals with alcohol consumption comparable to that of the MTBI group. Even if studies with this design should document traumatic lesions, it remains still uncertain whether lesions that are too discrete to be shown with a 1.0 T MR machine can explain the clinical symptoms of PCS.

## Competing interests

The authors declare that they have no competing interests.

## Authors' contributions

HS and DM conceived and designed the study, analyzed the data and drafted the manuscript. RG and SJ analyzed the MR and participated in the design and coordination of the study. DS participated in the study design and coordination and together with DM identified the patients in the study and control group. LJS participated in the design of the study, in analyzing the data and writing of the paper. DO participated in the design and coordination. All authors read and approved the final manuscript.

## Pre-publication history

The pre-publication history for this paper can be accessed here:

http://www.biomedcentral.com/1471-2342/9/11/prepub
